# Automated Spatial Omics Landscape Analysis Approach Reveals Novel Tissue Architectures in Ulcerative Colitis

**DOI:** 10.21203/rs.3.rs-3965505/v1

**Published:** 2024-03-11

**Authors:** Stephan Rogalla, Derek Holman, Samuel Rubin, Mariusz Ferenc, Elizabeth Holman, Alexander Koron, Robel Daniel, Brigid Boland, Garry Nolan, John Chang

**Affiliations:** Stanford University; Stanford University; Stanford University; Polish Academy of Sciences; Stanford University; Stanford University; Stanford University; University of California San Diego; Stanford School of Medicine; University of California San Diego

**Keywords:** spatial omics, computational analysis, inflammatory bowel disease

## Abstract

The utility of spatial omics in leveraging cellular interactions in normal and diseased states for precision medicine is hampered by a lack of strategies for matching disease states with spatial heterogeneity-guided cellular annotations. Here we use a spatial context-dependent approach that matches spatial pattern detection to cell annotation. Using this approach in existing datasets from ulcerative colitis patient colonic biopsies, we identified architectural complexities and associated difficult-to-detect rare cell types in ulcerative colitis germinal-center B cell follicles. Our approach deepens our understanding of health and disease pathogenesis, illustrates a strategy for automating nested architecture detection for highly multiplexed spatial biology data, and informs precision diagnosis and therapeutic strategies.

## Introduction

Ulcerative colitis (UC) is a gastrointestinal immune-mediated disease that occurs due to a combination of environmental stressors in a genetically predisposed individual. Endoscopically and histologically, UC is characterized by visible erythema, friability, and ulcers with inflammatory infiltrate. The clinical pattern of the disease itself presents with highly variable phenotypes, disease progression, and responsiveness to different therapies. Therapies targeting inflammation are modestly effective, but rates of primary non-response (13–40%) and loss of response (46% of the remainder) are relatively high[1–2]. Response rates could potentially be improved by more precise UC molecular subtype stratification[3–4].

Recent insights from spatial omics demonstrate that emergent properties from changes in tissue structural modules called architectures[5–6] are associated with disease subtypes, therapeutic response, and disease pathogenesis. This suggests that cell-type and tissue architectures serve as modular, functional building blocks. However, little is known about how changes in tissue structures are linked to disease initiation, progression, and therapeutic response. Improving our understanding of the role that architectural changes play in disease progression and recovery can enhance precision diagnostics and therapy by identifying potential functional relationships between patient therapy outcomes and their underlying tissue architectures. To accurately identify disease-associated cell-types and their relevant architectures, one must appropriately select the level of granularity for their cell annotations[7].

Spatial omics technologies historically applied to UC cell annotation were developed using analysis pipelines modified from dissociated single-cell analyses that lack explicit spatial information[8]. This critical step was determined by dimensional reduction and clustering, automatic cell-type identification based on a labeled reference from publicly available databases or datasets, or supervised learning[9]. Spatial considerations were only incorporated while validating cell identity by directly displaying those annotations on the multiplexed fluorescent image or when interrogating the spatial distributions of functional markers. Recent advances build on these historical approaches by incorporating spatial information such as neighboring cell types[10]. Their results, which concluded that classification accuracy is increased by taking neighboring cell types into account, suggest that biological architectures and spatial compartmentalization may also be promising candidates for refining cellular annotation. Another critical challenge in cell-type annotation arises from marker intensities that are not readily thresholded when presented in a dissociated-cell format[11]. Approaches that incorporate spatial considerations at the cellular level can address this challenge. By using patterns of spatial heterogeneities as a guide, marker intensities are evaluated in the context of specific, local architectures rather than the tissue as a whole. However, these new methods rely on deep learning technologies coupled with novel cell-type detection capabilities and may rely on manually annotated datasets[7]. Depending on the specific experimental or clinical question and because of its reliance on trained datasets, this approach may not adequately address the multiple levels of cell labeling details, also known as annotation granularity[7], necessary for a deeper understanding of a dataset across multiple spatial scales.

New technologies to identify and analyze architectures in tissues are crucial for a deeper understanding of the increasingly abundant publicly available spatial omics datasets. Many similar approaches have been pioneered in the geospatial sciences, including ecology and landscape ecology, as reviewed in Newman et al.[12]. Just as recent advances in spatial imaging technologies require the development of analytical tools and strategies for identifying, characterizing, and understanding tissue architectures and cellular interplay[5, 8, 10, 13], the development and proliferation of aerial photography in the 1930s inspired the development of tools for identifying and characterizing landscapes at multiple scales. These exploratory landscape analysis techniques offer insight into complex systems containing many spatially-resolved interacting components that interact across multiple scales to produce emergent properties. One critical parameter used in landscape analysis to represent the complexity of a system is physical entropy, which represents the spatially-resolved disorder in a system[12].

**Figure 1:**
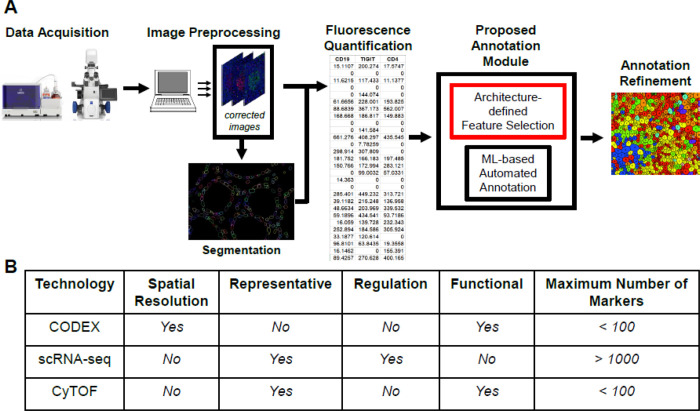
Proposed Implementation And Datasets. We propose to implement our add-on as a supplementary annotation module for existing annotation methods, including rapid, high-throughput ML methods (1A). Characteristics the of different single-cell technologies datasets used here (1B).

Since tissue architectures by definition represent compartmentalized function and chemistry, tissue architectures are therefore regions of decreased local entropy, or disorder. We anticipate that the quantification and ordering of multi-scale phenomena as addressed by hierarchical patch dynamics[14–15] and their emergent properties[16] will be widely used in spatial biology, especially once extended to multivariate parameter screens. Here we perform a proof-of-principle implementation of spatial metrics for the purpose of demonstrating the method’s applicability. We use spatial analysis by distance indices (SADIE) for pattern-dependent feature extraction based on observed randomness, regularity, or aggregation[17]. We generate both global and local metrics of randomness, regularity, and aggregation, allowing for architecture detection and visualization by thresholding as well as minimizing the need for assumptions regarding the spatial scale of cellular interactions. Here SADIE iteratively adjusts two-dimensional maps containing the x,y coordinates of individuals–in this case thresholded marker-positive cells, until the adjusted spatial distribution nears regularity. SADIE then assigns both global and local indices of aggregation summarizing the total distance individuals move[17–19]. We envision this strategy will be most effective for automated architecture-guided cell-type annotation, and should be implemented in formal workflows as a user-tuned add-on module that refines existing, rapid annotations by more generalized automated approaches (Fig. 1A)[10, 13].

Beyond label-transfer, we implement additional strategies for integrating spatially-resolved single cell technologies with existing single cell datasets. We integrate existing UC datasets from single-cell RNA sequencing (scRNA-seq), cytometry by Time of Flight (CyTOF), and Co-detection by Indexing (CODEX) datasets available in literature. The integration of these three technologies (scRNAseq, CyTOF and CODEX), each of which has distinctive yet complementary strengths and weaknesses (Fig. 1B), gives additional certainty to conclusions drawn from any one technology. Of the three technologies, only CODEX explicitly addresses **spatial** resolution. As shown by Zhao et al. [20], single tissue sections from a biopsy are unlikely to be statistically representative of that biopsy at scales ranging from the cellular to tissue levels. This consideration dominates when interrogating rare cell-types, rare architectures, and large but spatially complex architectures. Unlike CODEX, both scRNA-seq and CyTOF dissociate tissues into single cells and perform a random sampling prior to data acquisition. Aside from known differential cell sensitivity to the dissociation process, both scRNA-seq and CyTOF yield data that are statistically representative of the original biopsy[21–22].

CyTOF observations are more similar to CODEX in that both examine **functional** protein-level data while scRNA-seq provides **regulatory** transcript-level data. Integrating CyTOF and CODEX datasets can be challenging because of dimensional mismatch in the choice of observed parameters. Generally, there are relatively few (< 100) observed dimensions in comparison to scRNA-seq. These parameters are selected as an *a priori* panel. When datasets from different investigators are not specifically constructed to complement each other, the probability of marker overlap is low outside of core canonical cell markers[23–24]. In contrast to CyTOF and CODEX, scRNA-seq datasets often contain an order of magnitude (> 2000) greater observed dimensions. The probability of marker overlap is therefore increased. The well-known bias towards high-abundance transcripts in scRNA-seq poses an additional challenge[25]. We develop and demonstrate a workflow strategy that makes use of surrogate observations and superordinate architectures coupled with spatially-resolved per-architecture analysis rather than per-patient analysis to overcome the aforementioned dimensional mismatch challenges.

## Results

### Datasets

We used three publicly available single-cell resolution UC datasets–CODEX, CyTOF, and scRNA-seq– to demonstrate the effectiveness of our approach (Supplemental Table S1). The CODEX dataset was derived from colonic biopsies from 24 UC patients and 8 healthy HC patients[23]. The scRNA-seq dataset was derived from 10 HC and 10 UC patients, with samples from both tissue biopsies and patient blood[26]. The CyTOF dataset includes peripheral blood mononuclear cells (PBMCs) from 20 UC and 12 matched HC patients, as well as mononuclear cells from paired blood and colon tissue biopsy samples from an additional 12 UC patients [24]. Blood samples were classified as being from HC, UC-flare, or UC-remission. Inflamed biopsy samples were further matched to uninflamed control tissues from the same patient.

### Workflow

Our analytical workflow supplements canonical cell-type markers with B-cell follicle architecture-dependent features. We define a tissue landscape as “an area that is spatially heterogeneous in at least one factor of interest”[27]. Thus, our approach identifies marker-dependent spatial heterogeneities in cellular level representations within B-cell follicles. Architecture-dependent features are identified through a modified version of SADIE[17]. Once key cell populations are identified, we perform a secondary parameter screen for markers representing cell types that are indicative of architectures and their associated lower-granularity cell annotations. We assign increased weights to markers with high aggregation values (Ia) that are associated with only a small number of marker-positive cells (Supplemental Table S2), for clustering purposes.

**Figure 2:**
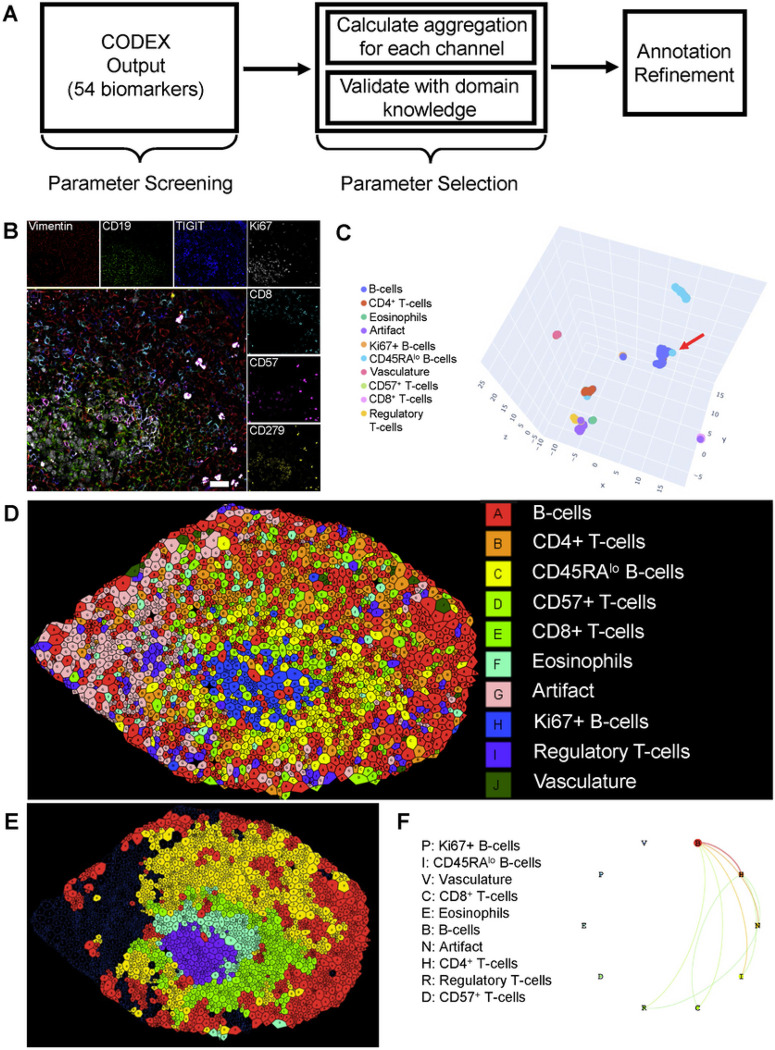
SADIE Analysis Identifies Rare Cell Types and Follicular Architectures. SADIE-guided workflow for cell annotation refinement, in which channels are initially screened in a quality control step. Aggregation values (Ia) are then obtained for each channel in the architecture of interest. High Ia-value channels are selected and supplemented with known canonical markers (parameter selection) for annotation refinement (2A). An example 7-channel fluorescent image of a proliferating UC B-cell follicle (2B). Scale bar = 300 μm. Note the honeycomb-like profile of membrane markers that results in segmentation difficulties and “bleed” into adjacent cells. Nonetheless, by directly incorporating spatial coordinates for further annotation refinement, we are able to re-annotate cells that appear to have been mis-clustered (2C, red arrow). Data space is visualized using a UMAP reduction (2C). The result of SADIE-guided parameter selection and subsequent annotation refinement is displayed on the associated Voronoi image, demonstrating the successful annotation even of rare cell types (2D). Associated cellular neighborhoods are displayed in their Voronoi representation (2E). Pairwise interactions, in which the size of each circle represents the fraction of all cells and the width of the line represents the thresholded ratio of interactions (2F).

By visualizing these screened parameters using scRNA-seq and CyTOF, we are able to characterize biological differences between UC and control (Fig. 2A).

### CODEX automated feature selection identifies complex architectures contributing to UC germinal center B cell follicles.

In order to simplify our analysis pipeline, we only selected cells that lay within follicle-associated regions of interest (RoIs) as determined by CD21 + cell aggregates visible in fluorescent images. Altogether, this process resulted in a total of 4 control and 51 UC-associated follicle RoIs (Supplemental Table S3), one of which is displayed in Fig. 2B.

Of a total of 53 fluorescent marker parameters, 13 were selected for cell identification based on canonical cell type markers, marker staining quality, and spatial distribution within representations of the follicles as assessed by SADIE. Spatial-associated parameters were selected based on their Ia values and statistical significance, weighted by the fraction of cells above the designated marker intensity value (Supplemental Table S1). By directly incorporating spatial coordinates and sequential sub-clustering steps into the clustering workflow followed by annotation refinement (Fig. 2C), we identify 10 different architecturally-relevant cell annotations (Fig. 2D), of which two are of particular interest. The first of these identities is CD56 + B cells which also express elevated levels of the established proliferation marker Ki67. This suggests that the CD56 marker on B-cells is either directly associated with proliferation or early stages of peripheral B-cell maturation. The second of these identities is a, to our knowledge previously unreported, rare cell population of CD57 + CD279 + CD4 + T cells (Fig. 2D) (Supplemental Figure S2) that lies exclusively within the proliferating center of germinal-center B cell follicles. We therefore select CD56 and CD57, Ki67-like proliferation markers (such as PCNA), and established markers of T-cell activation and exhaustion like CD279 for downstream validation in the representative technologies scRNA-seq and CyTOF, to support our CODEX observations. Although the rarity of these cells renders them difficult to detect using standard dissociated-cells single cell omics technologies analysis platforms–they comprise fewer than 1 in 50,000 total cells–the spatial resolution of CODEX renders them easily identifiable.

We then performed neighborhood and pairwise analysis to determine relevant architectural ‘zones’ within our follicle. Six such zones were determined (Fig. 2F), though one was discarded due to artifact proximity. The innermost zone, zone 1, is characterized by a core of proliferating Ki67 + CD56 + B cells. Zone 2 is similar to zone 1, but is interspersed with CD4 + CD279 + T cells, a small minority of which also express CD57. Zone 3 expresses higher levels of CD45RA relative to zone 1 and begins to lose CD19 expression while retaining CD21 expression. Zone 4 exhibits reduced numbers of CD4 + CD279 + T cells, and we further begin to observe the presence of eosinophils and Foxp3 + T regulatory cells. Finally, zone 5 is marked by the encroachment of CD8 T cells. Together, these results suggest that germinal center B cell follicles in UC are highly complex architectural structures with numerous zones of development and maturation that are regulated by numerous different classes of T cells.

### scRNA-seq verifies changes in proliferating B cells and associated T cell phenotypes

**Figure 3:**
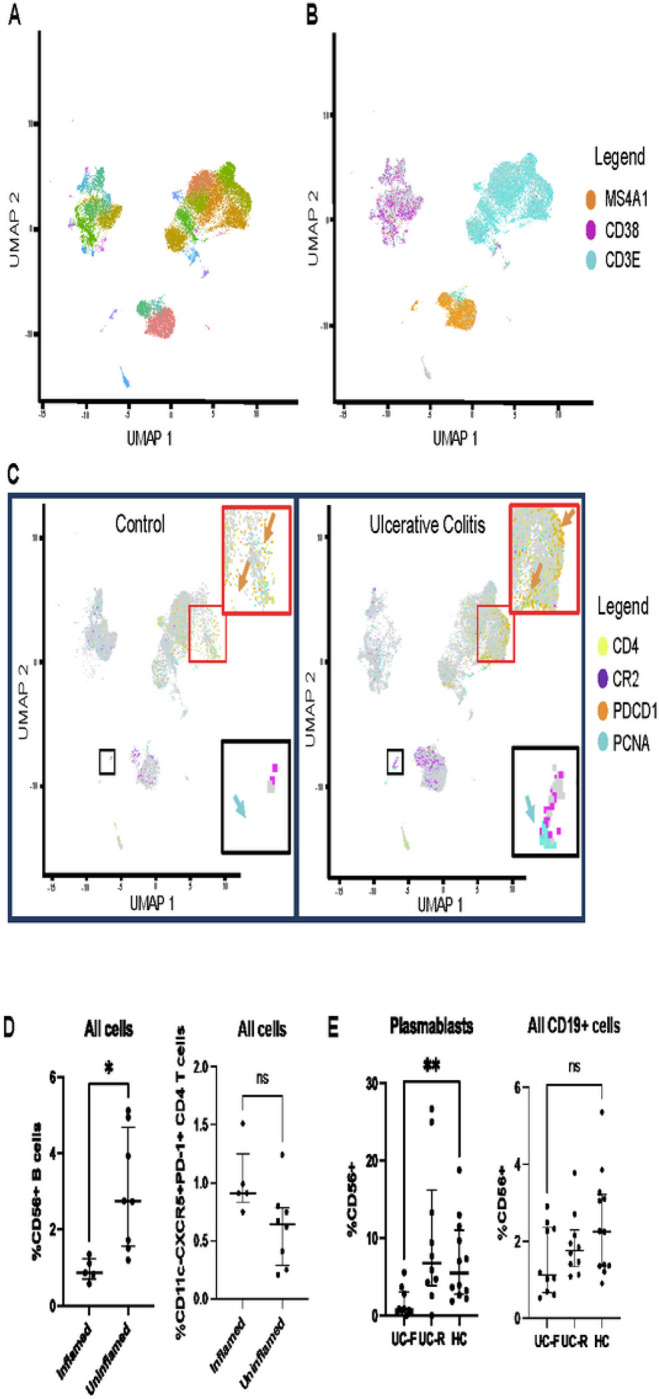
Evaluation of CODEX Results using scRNA-seq and CyTOF Initial clustering results for scRNA-seq of colonic biopsies (3A). UMAP representation and visualization of canonical markers identifies primary topographical features of interest: MS4A1 is a B-cell marker, CD3E is a T-cell marker, CD38 is a plasma cell marker (3B). CD4 and PDCD1 (CD279) are displayed in the T-cell cluster (3C, red inset). Orange arrows indicate the PDCD1+ CD4+ T-cell region in UC samples (3C, red inset, right), as well as the corresponding depleted region in healthy controls (3C, red inset, left). CR2 (CD21) and PCNA (Ki67 surrogate) are displayed in the proliferating B-cell cluster (3C, black inset). Blue arrows indicate the PDNA+ area of the MS4A1+ B-cell island in UC samples (3C, black inset, right) as well as the corresponding depleted region in healthy controls (3C, black inset, left). CyTOF reveals CD56+ B-cells and exhausted CD4 T-cells in UC patient colonic biopsies, though adjacent uninflamed controls have elevated proliferating B-cells (3D). CD56+ B-cells as a fraction of all plasmablasts are reduced in blood during disease flare, relative to remission and healthy control (3E), though similar differences are not observed as a fraction of all CD19+ cells.

Due to the low number of visibly (Ki67+) germinal center B cell follicles in the CODEX dataset relative to the number of tissue sections, we confirmed the increased presence of germinal center B cell follicles and potential follicular T cell subsets using scRNA-seq. When visualized using UMAP using gold standard approaches[28–29], we are able to identify clusters corresponding to visually evident topographical features (Fig. 3A). Broadly, cells readily separate into three primary groups: plasma cells, T cells, and B cells (Fig. 3B). We then proceeded to map our candidate CODEX markers onto our scRNA-seq dataset visualization.

Critically, neither of the transcripts associated with candidate markers Ki67 and CD57 were present in the scRNA-seq dataset. Instead we used cell division-associated PCNA as a surrogate marker for CD56/Ki67, and PDCD1 (CD279) as a marker for the parent cell population of our CODEX CD4 + CD278/279 + CD57 + population (Fig. 3C). When visualized using UMAP topography, none of these markers were well-captured in their own clusters nor during subsequent subclustering. This indicates that gold-standard scRNA-seq analysis methods would have failed to identify these populations as being different between UC and control. This consequence reflects both limitations on data analysis as well as sequencing depth. However, because these markers are indicated as being spatially-segregated components, we are able to selectively visualize them in our scRNA-seq dataspace. We can therefore conclude that these cell types are increased, in the case of the CD56+/Ki67 + B cell population, and that the permissive environment of parent cell populations is increased, in the case of the CD4 + CD278/279 + CD57 + T cell population, in UC patients.

### Mass cytometry verifies mucosal immune dysregulation in UC

When using CODEX, we observed that germinal-center B cell follicles predominantly existed in tissue sections from inflamed biopsies, although the proliferative center was only visible in a small subset of sections. Our scRNA-seq analysis confirmed the elevation of these architectures in UC versus healthy control biopsies. Similarly, when examining the CyTOF dataset, substantial numbers of CD56 + B-cells were observed in UC patients indicating the presence of germinal center follicles, even though direct proliferation-associated markers were not included in the panel. Unexpectedly, these cells were even more abundant in adjacent, uninflamed biopsies from UC patients (Fig. 3D). This appears to suggest that adjacent uninflamed biopsies are fundamentally different from biopsies from healthy controls and may retain disease-associated B-cell dependent architectural motifs. Exhausted CD4 + T-cells, not present in appreciable numbers in healthy controls, were similarly observed to be present in both inflamed and uninflamed UC biopsies. While the depletion of CD56 + cells from blood is observed during UC flare (Fig. 3E), suggesting that flare may involve additional trafficking from the blood to local sites of inflammation, these cell types do not appear to be represented by a dedicated cluster in our scRNA-seq analysis of blood samples as they were in our analysis of colonic biopsies (Supplemental Figure S2). Furthermore, these cells lack proliferation-associated markers.

## Discussion

Spatial omics hold great promise for precision medicine due to the possibility of linking multiscale cellular interactions to aggregate functions that may underlie disease heterogeneity. However, architecture detection is sensitive to cell annotations, especially with respect to annotation granularity, and there is a lack of methods that directly incorporate spatial heterogeneities in cell-type annotation. Here we show in Ulcerative Colitis (UC) that combining computational landscape ecology methodologies with spatial omics enables the ability to extract rare cell types. This approach is especially useful for cells that occupy restricted spaces within larger architectures such as germinal-center B-cell follicles that are likely to be of importance but would be otherwise missed due to their spatial distribution. We developed an automated pipeline to optimize existing cell annotation strategies for the characterization of tissue architectures. We demonstrate its success for increasing confidence in imaging results and bypass random-sampling weaknesses through the incorporation of existing, publicly available data from other single-cell omics technologies.

Our method enables spatial feature-dependent annotations that were previously co-classified into overarching architectural categories[23]. We demonstrate that our method gives additional statistical certainty to conclusions regarding tissue architectures in UC from spatial omics technologies. We also demonstrate that by applying our method to CODEX we discover and localize rare cell types in UC patient biopsies. By leveraging the conditional spatial distribution of these cells within permissive, superordinate architectural structures that are more easily visualizable using scRNA-seq, we are able to conclude that these rare cells are not present in healthy control biopsies. The ability to assess both cell type supersets as well as cell architecture supersets for rare cell types provides a powerful advantage over dissociated-cell technologies. Because of the lack of explicit spatial information, although some spatial information may be implicitly encoded even in dissociated cells because of proximity-dependent changes to transcriptomes[30], dissociated-cell technologies primarily allow assessments only through cell type supersets.

Using these improved annotations, we obtain new insight into the spatial intricacies of peripheral colonic B-cell follicles in UC colonic biopsies. We identify two rare cell types in addition to five architectural components in these follicles. While the function of the first rare cell type is unknown, the spatial restriction of these T-cells to the follicle germinal center suggests a regulatory role associated with B-cell maturation. The second rare cell type, an immature, proliferating follicle-associated B-cell population, shares markers with a subpopulation of blood-associated circulating B cells. This suggests that this fraction of circulating cells may ultimately migrate into the colon to form large, germinal center B-cell follicles, consistent with recent reports[31–32]. Although B cells play key roles in the pathophysiology of UC across peripheral, central, and mucosal immunological compartments, B-cells are relatively understudied in IBD compared to T-cells and myeloid lineage cell populations[32–33].

While B-cells have not been uniquely targeted by the approved IBD therapies, B-cells are significantly affected by current treatments and are putative targets for future IBD therapies[33]. Additional characterization of these structural components can potentially be used to develop therapies that modulate germinal center B-cell follicles in UC. We also observe differences between our healthy patient controls and our uninflamed tissues from UC patient controls, suggesting that regions designated as uninflamed may carry either residual disease-associated tissue architectures or architectures that are primed for disease flare. The formation and persistence of these architectures is potentially linked to the cycles of flare and remission commonly encountered by UC patients as reviewed in Lissner et al.[34].

Our approach should be useful for architectural interrogation, with the goal of enabling precision medicine by linking tissue architectures with disease phenotype or therapy response. While there are limitations, the majority can be mitigated through careful data collection, processing, and analysis workflows. One area that should be addressed in future implementations involves the approach used for marker thresholding. The thresholding approach assumes that marker intensities are readily thresholded; this assumption may not hold true if markers exhibit a gradient or multimodal intensity distribution. Using our CODEX dataset’s CD56, for example, although a subset of B cells were clearly CD56 positive, the fact that CD56 intensities were much higher elsewhere rendered single-threshold determination ineffective for architectural feature detection (Supplemental Figure S3).This limitation can be addressed through the use of sliding spatial windows to determine spatial patterns in overall intensity represented at the cellular level. Once the different spatial patterns are identified, each channel can be split into pseudo-channels, in which each pattern can be treated as its own marker channel for the purpose of assessing feature selection by automated methods.

Our results demonstrate the power of directly leveraging marker spatial heterogeneities for automating cell-annotation algorithms, for the detection of rare cell types, and for integrating different large datasets even when direct label transfer is not possible. As spatial omics technologies become able to assess thousands or tens of thousands of markers, manual annotation-refinement becomes formidably labor- and time-intensive[13]. With rapid advances in functional imaging, our automated approach should be able to serve as a key part of the pipeline for identifying and characterizing modular tissue architectures, as well as predicting how they locally contribute to the spectrum of health and disease.

## Materials And Methods

### Data set acquisition

CyTOF and segmented and fluorescence-assigned CODEX datasets were acquired from the original authors [23, 24]. The scRNAseq dataset was downloaded from GEO [26].

### CODEX Follicle Extraction

Follicle ROIs were identified in FIJI based on CD19 + staining aggregation in the original images. Cell centers within each ROI were extracted in R, using the original .csv fluorescent intensity text file as well as the follicle-determined spline exported from FIJI to rapidly determine which cells were within the ROI and which were outside the ROI.

### CODEX Data Pre-processing

Manual inspection of fluorescent image channels revealed that imaging “artifacts” were derived from two primary sources: eosinophils, which exhibited broad cytoplasmic binding to the majority of antibodies but membrane-staining for CD15 and CD66 and smaller, punctate sources that persisted across the majority of channels. True (non-eosinophil) artifacts were identified based on broad, high-level expression of many markers except CD15 and CD66; cell center positions were retained but all marker intensities were set to 0. Due to high expression of many markers including membrane-stain signatures for CD15 and CD66, eosinophils were identified then temporarily removed from analysis. Residual artifact impacts, likely derived from segmentation, were identified by selecting a representative channel with minimal signal other than artifact, determining average signal within a non-artifact-impacted stripe, flagging all cells with signal higher than three times average background, then setting all marker intensities in flagged cells to 0.

### SADIE Analysis

Per fluorescent channel and using a threshold of 0.33, all cells with fluorescent signal exceeding 0.33*max were set to a value of 1 (marker-positive); all others were set to a value of 0 (marker-negative). SADIE analysis was then performed once for each fluorescent channel, coupled with the cell center-associated x and y coordinates, using the epiphy package implementation of SADIE and the following parameters: index = “Perry”, nperm = 100, method = “shortsimplex”, verbose = TRUE.

### CODEX Feature Selection

After SADIE analysis, comparing the spatial distribution of marker-positive cells to 100 random distributions using the same number of marker + cells (Perry), a core set of canonical cell markers was supplemented by markers exceeding an Ia threshold of 2.0 or a Pa value of 0.05 (Supplemental Table S2).

### CODEX Cell Clustering

Initial cell clustering was performed in R, using the built-in k-means function. The initial number of clusters was determined using the fviz_nbclust implementation of elbow plots. After normalization, architecture-associated features that were marker-positive in fewer than 25 cells were given additional weight. Clusters were then displayed on the original multiplexed fluorescent image for validation using code developed by Goltsev et al. [8], with parameters tuned accordingly.

### CODEX Cell Annotation Refinement

Cell annotation refinement was performed using in-house software developed by M. Ferenc.

For the purposes of this study we built meaningful vector spaces with which we could observe well-separated clusters. Briefly, data were encoded using a one-hot approach. Principal Component Analysis was then used to generate a latent space. Finally, data clusters were visualized using UMAP for annotation refinement.

Initial annotations for clustering were compiled into a single matrix per follicle in which each row is a single cell and the columns correspond to feature-extracted arcsinh-transformed marker fluorescent intensities, initial cluster ID, X, and Y coordinate. For initial data preprocessing, in order to address imbalances in dataset cluster sizes (Supplemental Figure S4) 150 cells were randomly sampled from each cluster.

Model training was performed using a basic MLP (Multi Layer Perceptron) model with an input of 15 neurons, a latent space of 16 neurons, and an output layer of 10 neurons. We performed 5-fold cross validation with early stopping to avoid model overfitting. Cross-validation scores are shown in (Supplemental Table S4).

Using the MLP’s dense layer to visualize its final transformation effect, we then generated visualizations of its functional vector space based on the model’s latent space separating 10 cluster types. Latent representations were visualized after applying PCA, UMAP, and t-SNE for dimensionality reduction to 3 dimensions. Final annotation refinement was performed based on the UMAP visualization’s improved cluster separation.

### Generation of Voronoi images

Voronoi diagrams were created using custom code developed in Goltsev et al. [8].

### Identification of CODEX Neighborhoods

For each cell in the follicle, the 10 nearest cells, including the original cell, were determined based on the annotations from CODEX cell clustering and refinement. The composition of these microenvironments was clustered using X-shift clustering with supervised annotation, using publicly available software from the Nolan lab Github. Neighborhood annotations were then re-displayed in Voronoi images.

### CODEX Secondary Feature Selection for cross-platform comparison

Features for cross-platform comparison were selected based on association with architectural sub-structures. In the event that these features were not present in the CyTOF or scRNA-seq datasets, functionally similar markers were used instead–for example, PCNA instead of Ki67. Where this was not possible, we used markers that demonstrated spatial colocalization, either directly or as a superset.

### scRNAseq analysis

The R package Seurat [35] was used for analysis of scRNA-seq datasets, which had already been subjected to standard pre-processing [26]. The selection of initial parameters was guided using elbow-plots, with an initial clustering resolution of 1.5, then further tuned based on the visualization of canonical markers on UMAP featureplots .

### CyTOF analysis

Processed CyTOF data were obtained from the original authors and analyzed as previously described [24]. In brief, FlowJo software was utilized to gate cellular events and calculate statistics according to published conventions, and GraphPad PRISM 9 was utilized for conducting additional statistical tests and plotting figures. P-values were computed by unpaired Student’s T-test.
